# Investigating conceptions of intentional action by analyzing participant generated scenarios

**DOI:** 10.3389/fpsyg.2015.01630

**Published:** 2015-11-05

**Authors:** Alexander Skulmowski, Andreas Bunge, Bret R. Cohen, Barbara A. K. Kreilkamp, Nicole Troxler

**Affiliations:** ^1^E-Learning and New Media, Institute for Media Research, Technische Universität ChemnitzChemnitz, Germany; ^2^Department of Philosophy, University of NottinghamNottingham, UK; ^3^Institute of Cognitive Science, University of OsnabrückOsnabrück, Germany; ^4^Department of Molecular and Clinical Pharmacology, Institute of Translational Medicine, University of LiverpoolLiverpool, UK; ^5^Institute of Psychology, University of OsnabrückOsnabrück, Germany

**Keywords:** intentional action, experimental philosophy, Knobe-effect, concepts, lay conceptions, content analysis

## Abstract

We describe and report on results of employing a new method for analyzing lay conceptions of intentional and unintentional action. Instead of asking people for their conceptual intuitions with regard to construed scenarios, we asked our participants to come up with their own scenarios and to explain why these are examples of intentional or unintentional actions. By way of content analysis, we extracted contexts and components that people associated with these action types. Our participants associated unintentional actions predominantly with bad outcomes for all persons involved and linked intentional actions more strongly to positive outcomes, especially concerning the agent. People’s conceptions of intentional action seem to involve more aspects than commonly assumed in philosophical models of intentional action that solely stress the importance of intentions, desires, and beliefs. The additional aspects include decisions and thoughts about the action. In addition, we found that the criteria that participants generated for unintentional actions are not a mere inversion of those used in explanations for intentional actions. Associations between involuntariness and unintentional action seem to be stronger than associations between aspects of voluntariness and intentional action.

## Introduction

Due to their key importance for social understanding, ascriptions of intentionality are an intensely studied research topic within the cognitive sciences, in particular in the fields of social cognition (Malle et al., 2001) as well as in philosophy of mind and action (Bratman, 1987; Audi, 1997; Mele, 2009). In recent years, there has been a clear tendency toward combining empirical research and armchair reflection in order to investigate philosophical topics that were previously mainly discussed on a purely theoretical level. This newly risen appreciation of empirical methods in philosophy has spawned the so-called experimental philosophy movement (Weinberg et al., 2001; Knobe, 2003a; Machery et al., 2004; Knobe and Nichols, 2008). While analytical philosophers try to understand the meaning of a concept by thorough reflection on its essential components, experimental philosophers employ methods from the social and cognitive sciences to examine the use of a concept. They investigate what conceptual intuitions people have, i.e., to which cases they apply a particular concept, and they attempt to provide an account of the internal psychological processes that underlie such applications (Knobe and Nichols, 2008). Most experimental philosophy research takes the form of surveys in which participants are confronted with various textual scenarios (sometimes also called vignettes) and asked whether they would apply the concept in question. It is frequently claimed that the insights from this research are not only interesting in their own right, but that they also provide valuable input for philosophical debates on the meaning of particular concepts.

In this paper, we focus on the lay understanding of intentional (and unintentional) action and describe how we have applied a new method for analyzing it. We avoid speaking of the (folk) *concept* of intentional action, unlike other researchers (e.g., Malle and Knobe, 1997). For at least two reasons it is very controversial whether, and if so to what extent, experimental philosophy studies can actually inform us about conceptual content. First of all, there is no agreed definition of ‘concept,’ let alone ‘conceptual content’: depending on different theoretical commitments, concepts may be viewed as mental representations of various kinds, as abilities, or as Fregean senses; and they may be seen, amongst others, to have definitional, prototypical or theoretical structure (Margolis and Laurence, 2014). Secondly, the application of a concept may partly be driven by other factors than the content of the concept, such as pragmatic norms or currently present emotions (Machery, 2008). Although it is disputable to what extent experimental philosophy research informs about the meaning of concepts, it seems uncontroversial that it can fulfill two important functions. Firstly, it can inform us about what factors influence the application of certain notions, such as the notion of intentionality, to particular cases. This is achieved by the usual approach of presenting participants with scenarios varying in one factor and to asking them for their conceptual intuitions with regard to these scenarios. Secondly, experimental philosophy research can inform us about how non-philosophers understand certain notions such as ‘doing something intentionally.’ This can be achieved by asking participants open questions about how they understand the notion in question. Both of these approaches have advantages, but also limitations. The scenario approach allows an examination of the influence of specific factors on conceptual judgments in a very controlled manner. However, by examining single factors, it is difficult to assess the relevant importance of the different factors that may play a role in the conceptual judgement. Moreover, scenario studies may run the risk of missing important factors. The advantage of open questions is that they can bring factors into focus that have been neglected in previous scenario-based research and that the responses to the questions may allow the assessment of the comparative importance of the factors that play a role. The downside of this approach is that people’s responses to open questions may reflect (more than the scenario approach) different aspects, such as the actual meaning of a notion, the pragmatic norms governing the use of the notion, psychological factors that influence the application of the notion, or lay theories about the usage of the notion, and these aspects may be difficult to disentangle. In this paper we follow the second approach, because we think it is still an underexplored method in experimental philosophy, but we acknowledge that both approaches are important and work best in concert. Next, we will briefly review the previous experimental philosophy research on intentional action in order to further motivate our open question approach.

A study by Knobe (2003a) had a considerable influence on the experimental philosophy movement as we know it today. He used the now famous ‘chairman’ scenario in order to examine under which conditions people would say that the side effect of an action was brought about intentionally. The scenario involves the chairman of a company who needs to decide whether a business project that would increase profits but also harm the environment, should it be implemented. He decides to carry out the project, saying that he does not care at all about harming the environment and that he wants to make as much profit as he can. Knobe found that most people thought that the chairman harmed the environment intentionally. In an alternative version that described exactly the same situation as the first one, with the exception that the word ‘harm’ was replaced by the word ‘help,’ most people did not believe that the chairman helped the environment intentionally. This asymmetry in judgment is now known as the *Knobe effect* or the *side-effect effect*. Knobe also found a corresponding asymmetry in people’s assignment of praise and blame, namely that they are considerably more willing to blame the agent for bad side effects than to praise the agent for good side effects. Knobe’s study provoked a host of explanations for the asymmetry, as well as descriptions of mechanisms hypothesized to be involved in intentionality ascriptions in general (for reviews, see Feltz, 2007 or Cova, forthcoming). We shall briefly review some of these.

Adams and Steadman (2004) argued that pragmatic features of the term ‘intentionally’ can explain Knobe’s surprising findings. When people say that someone did something intentionally, they often use this term in order to assign blame. Consequently, the intentionality ascriptions in Knobe’s study were influenced by people’s desire to blame the chairman for harming the environment. In accordance with this interpretation, Guglielmo and Malle (2010) found that when people had the possibility of choosing between different descriptions, a vast majority find the phrase ‘the chairman knowingly harmed the environment’ most accurate, while only a few participants choose the phrase ‘the chairman intentionally harmed the environment.’ Uttich and Lombrozo (2010) added a different interpretation, according to which adherence to and violation of norms influence intentionality attributions. In contrast, Phelan and Sarkissian (2008) showed that side effects in scenarios that include neither a morally significant action nor the violation of any kind of norm are still rated as brought about intentionally. According to Machery (2008), people think in terms of costs to be paid, whereas according to Sripada (2010), people take the central character traits of an agent into account when determining whether she did something intentionally.

These are just a few examples from the extensive experimental philosophy literature on intentionality ascriptions. The point we want to stress is that all these studies use a similar methodology that we deem incapable of exhaustively explaining the use of the notion of intentionality. They predominantly rely on scenarios that vary one component over different conditions in order to gain insights into the influence of an isolated aspect on the ascription of intentionality. While offering a clear and valid experimental treatment with which the influence of certain, isolated factors on intentionality ascriptions can be measured, the insights that can be gained through this method are limited and may not explain the whole picture. In the particular case of the Knobe scenarios, there is an additional methodological problem lowering the validity of the studies. Methodological research has revealed that studies consisting of rare or implausible scenarios are likely to distort responses, as participants disregard, or fully exclude, the aspects causing the implausibility from their decisions: in a paper by Auspurg et al. (2009) the influence of implausible and uncommon scenarios on conceptual responses was investigated, revealing that participants may take such scenarios less seriously. They presented their participants with a series of different short descriptions of the educational level, occupation, and demographic information of fictional persons and asked whether the income of that person was justified, in order to assess which of these dimensions participants include in their judgments. Among other variations, some of the scenarios were rare or implausible, e.g., describing a person that works in a field that normally requires higher education or training, but who did not obtain this kind of education. With the first presentation of an implausible case, responses changed, even affecting scenarios presented after the implausible ones. This result was interpreted as an indication of the participants’ tendency to take a study less seriously as soon as an implausible case was presented, posing a serious threat to the validity of results obtained from such scenarios. Indeed the behavior of the chairman in Knobe’s scenario is likely to be perceived as odd. In our contemporary society, openly admitting not to care about helping the environment and to wanting to make as much profit as possible would be seen as unusual. After all, the chairman could just remain silent about his indifference about helping the environment. Due to this unusual scenario in which the agent presents himself in a more negative way than necessary, we claim that the scenario only appears to be a plausible, everyday situation, but on closer inspection turns out to be a very rare case. Consequently, the validity of the judgments made regarding the Knobe scenarios may be lowered, casting doubt on their generalizability to other situations and most importantly, whether this case is able to shed light on processes involved in everyday intentionality ascriptions. Moreover, most of the experimental philosophy studies have in common that they do not take into consideration what people themselves believe to be relevant for an action to be intentional.

Knobe’s above-mentioned study from 2003 has strongly influenced the methodology that experimental philosophers have employed since then. Ironically, a few years before that study, Malle and Knobe (1997) had published an article in which they reported a survey (study 2 in that paper) that employs a method which we deem more capable of revealing people’s general conception of intentional action. In that study, they asked people explicitly what they have in mind when they say that somebody did something intentionally. They then analyzed the participants’ written definitions in order to determine to which aspects they refer. Malle and Knobe’s straightforward approach has the clear advantage that it takes into account ordinary people’s ideas about intentionality. Setting aside the dispute about whether intentions can be reduced to sets of desires and beliefs, there is a wide agreement among philosophers of action that intentions, beliefs, and desires are closely linked to intentional action (e.g., Bratman, 1987; Audi, 1997; Malle, 2004; Mele, 2009). It is usually assumed that an agent who acts intentionally desires a particular outcome, believes that the action will produce this outcome, and consequently intends to perform the action. Moreover, it has been proposed that one needs to believe that one is able to perform the action and to bring about the desired outcome in order to act intentionally (Shaver, 1985). Consistent with this broad consensus, Malle and Knobe identified desire, belief, and intention as dominant components in their participants’ responses. However, in contrast to common three-way models of intentional action, they identified awareness as a fourth essential component in the given definitions. Moreover, although not mentioned by the participants in their explanations, an additionally conducted scenario study (study 3 in that paper) led them to conclude that the agent’s skill in performing the action adds a fifth essential component to people’s concept of intentionality. However, in a later article, Knobe (2003b) rejected the view that skill is always essential. In a series of studies he showed that evaluative considerations have a significant influence on whether people consider skill to be crucial for acting intentionally. For instance, people tend to judge immoral actions to be intentional, even when the agent has demonstrably no skill to perform the action.

Although we agree with Malle and Knobe’s general approach of employing open questions, we remain skeptical about their specific method of data analysis. The categories to which they assigned participants’ responses were very broad. They grouped a variety of vastly different statements under each of their categories. For instance, the intention category included ‘the intention to perform the act’, ‘intending,’ ‘meaning,’ ‘deciding,’ ‘choosing,’ and ‘planning to perform the act.’ It might well be true that all these terms can be analyzed as being related to intention. For example, we may define a decision as an act of intention formation and planning can be seen as intention guided action preparation. However, even if we assume that these are uncontroversial conceptual analyses of ‘decision’ and ‘planning’ (which is disputable), Malle and Knobe run the risk of eliminating potentially important data from their analysis when they lump these terms together. After all, it cannot be disputed that decisions, plans, and intentions, although conceptually related, are different phenomena. It might be the case that participants deem decisions or plans to be important for intentional action irrespective of their (possible) relation to intention. Using very few, theory-driven categories to analyze participant generated responses is a common approach, because it simplifies the coding procedure (see also Monroe and Malle, 2010; Stillman et al., 2011; Böhm and Pfister, 2015), but in our view, these classifications are too strongly driven by the researchers’ preconceptions rather than by participants’ actual responses. Our aim is to develop a method of analyzing explanations in a manner that stays closer to the actual responses.

We may distinguish two ways of analyzing participant generated responses: confirmative and exploratory analysis. A confirmative analysis uses free responses in order to test specific theoretical assumptions about how people apply or understand certain notions. Malle and Knobe’s (1997) study, as well as the other studies mentioned in the last paragraph, can be seen as belonging to this category. In these studies broad categories are applied to the data to test hypotheses that are, although not always explicitly, derived from previous theorizing. Another approach is an exploratory analysis that seeks to impartially derive categories from the actual responses given. Such an exploratory analysis resonates with the Grounded Theory approach, which puts emphasis on deriving theories from qualitative data, rather than just using the qualitative data to verify existing theories (Glaser and Strauss, 2009). We argue that a confirmative analysis should ideally be preceded by an exploratory analysis, so that components that are not already included in existing theories can be found and evaluated. In the present study we will pursue such an exploratory strategy, since we want to allow for the possibility that factors that have not been included in philosophical models of intentional action turn out to be important in lay conceptions of intentional action.

We see our study as contributing to the development of new methods that are able to reveal the components of abstract knowledge structures more accurately (see also Bunge and Skulmowski, 2014). We think that people may find it difficult to answer a general definitional question, such as the one posed by Malle and Knobe (1997). They asked ‘When you say that somebody performed an action intentionally, what does this mean? Please explain.’ The notion of intentional action is rather abstract and the majority of people will not have a satisfactory definition readily at hand if asked for one. Based on empirical evidence indicating the central role of concrete experiences in the formation of knowledge about abstract entities (Barsalou, 1999, 2005), we assume that providing examples of concrete intentional actions will make it easier to give a definition of intentional action. The concrete examples will facilitate the recall of important information that would otherwise be rarely available. Instead of asking people what they mean when they say that somebody did something intentionally, we asked the participants of our survey to come up with several concrete examples of intentional and unintentional actions or behavior, including a short description of the specific situation they have in mind. The underlying assumption of this approach is that people’s conception of intentionality determines which kind of examples they come up with. These examples eventually allowed us to determine, by means of content analysis, some contextual factors that people associate with intentional and unintentional actions. We also provided the participants with the opportunity to indicate how sure they were about their example being an instance of an intentional or unintentional action. Furthermore, we asked them to explain why they believed that each particular action described was intentional or unintentional. From the analysis of these explanations, we attempted to determine what criteria people think are constituents of intentional and unintentional actions.

A prominent criticism of experimental philosophy is that surveys can only inform us about surface intuitions, whereas philosophical dialog and reflection is needed to reveal robust intuitions (Kauppinen, 2007). In order to deal with this criticism, we designed our survey in a manner that prevents participants from being rather passive “evaluators” of a given scenario, instead letting them actively describe their associations and justify their explanations, coming closer to a philosophical dialog than the current standard method. By asking our participants for several examples of intentional and unintentional actions, by requesting assessments of their degree of certainty, and by requiring them to provide explanations, we prompt the participants to actively reflect upon the notion of intentional and unintentional action, and to refine their responses during this process. As each participant comes up with several scenarios, the risk that only very atypical examples are mentioned is minimized. Typical examples have a higher probability of being recalled and produced (Rosch, 1978). This ensures that the numerical distribution of criteria that are extracted from the explanations adequately mirrors the relative importance of each of these criteria within the lay conception of intentional action.

We decided to focus our attention on three hypotheses. While the first hypothesis targets the scenarios of (un-)intentional action, the second and third hypotheses refer to the participant’s explanations for why a given action is (un)intentional. Firstly, we assumed that doing something intentionally is associated with positive outcomes, while unintentionality is linked with negative outcomes (Hypothesis 1). This expectation might seem surprising in the light of the Knobe effect. In fact, it is the opposite of what we would expect if the Knobe effect was generalizable to intentionality ascriptions *per se*. In Knobe’s (2003a) study an agent who brought about a negative consequence (harming the environment) was predominantly judged to act intentionally, while an agent who produced a positive outcome (helping the environment) was largely judged to act unintentionally. We suspect that this effect is generated by the specifics of the scenario used. Commonsense tells us that intentional actions are not necessarily harmful. Admittedly, people sometimes intentionally harm others or their environment, but for most people this is not the standard mode of action. Quite to the contrary, most of our everyday intentional actions are aimed at producing positive outcomes for ourselves or those people around us. We expected that our participants’ responses would reflect this assumption. By contrast, unintentional action may well be associated with negative outcomes, as they are not under people’s control. In fact, we often excuse negative outcomes by saying that we did not produce them intentionally. As a result, we expected that our participants’ unintentional action scenarios would predominantly feature negative outcomes. In line with our exploratory approach, we did not formulate any specific predictions as to what criteria our participants would mention in their explanation for why a certain action is intentional or unintentional. However, we had a general expectation that those criteria that our participants would mention would be more finely differentiated than those components (belief, desire, intention) ordinarily highlighted in philosophical models of intentional action (Hypothesis 2). As far as we are aware, no one else has so far investigated in detail the nature of people’s conception of *un*intentional action. The tacit assumption seems to be that the lay conception of unintentional action is just an inverted version of the lay conception of intentional action. By contrast, we expected that people would refer to different, and not just inverted, criteria in their explanations for why a given behavior is intentional, as compared to those criteria mentioned in the explanations for why a behavior is judged to be unintentional (Hypothesis 3).

## Materials and Methods

### Participants

The study was conducted as an online survey. The link was distributed over social networks and online forums. A coupon lottery was used as an incentive to participate. Only native English speakers over the age of 18 were invited to participate, in order to keep the results comparable with results of existing research. Over a period of 3 months, 131 participants (84 female) completed the survey. Informed consent was obtained from all participants. The study was approved by the ethics committee of the University of Osnabrück.

### Experimental Procedure and Materials

After giving their informed consent and confirming that they are native English speakers, participants indicated their gender. The first part of the survey consisted of three pages with the following instructions and questions on each page:

(1) Please come up with one scenario of a specific person (John) doing something intentionally. Please also describe the setting. (2) How sure are you that your scenario is an example of doing something intentionally? Please rate on a scale from -3 (completely unsure) to 3 (completely sure). (3) Why do you think what he does in your scenario is intentional? Please explain. These items were followed by three further pages, each containing the following instructions and questions:

(1) Please come up with one scenario of a specific person (Paul) doing something unintentionally. Please also describe the setting. (2) How sure are you that your scenario is an example of doing something unintentionally? Please rate on a scale from -3 (completely unsure) to 3 (completely sure). (3) Why do you think what he does in your scenario is unintentional? Please explain.

As each participant accordingly provided us with six scenarios, we ended up with a total number of 786 scenarios, each with a corresponding explanation. Half of these (393) were scenarios featuring intentional actions or behavior, while the other half consisted of scenarios with descriptions of unintentional actions or behavior. It is to note that we use the terms ‘action’ and ‘behavior’ interchangeably, because the focus of our research is on how people distinguish between intentional and unintentional action/behavior, rather than on how they distinguish between action and mere behavior.

### Scenario Categories

In the case of the scenarios, we agreed that two dimensions were of particular interest. Firstly, although not directly bearing on any of our hypotheses, we judged it worthwhile to record whether or not other people beside the agent were involved in the described scenarios. Scenarios in which only the agent (John or Paul) was involved were labeled as ‘non-social,’ whereas scenarios in which others were also involved were labeled as ‘social.’ We were interested in whether there is a difference between the relative degree of association of each of these two cases with intentional and unintentional actions. The concrete definitions of these and the other categories mentioned below were listed in the coding instructions that were handed to the coders.

**FIGURE 1:**
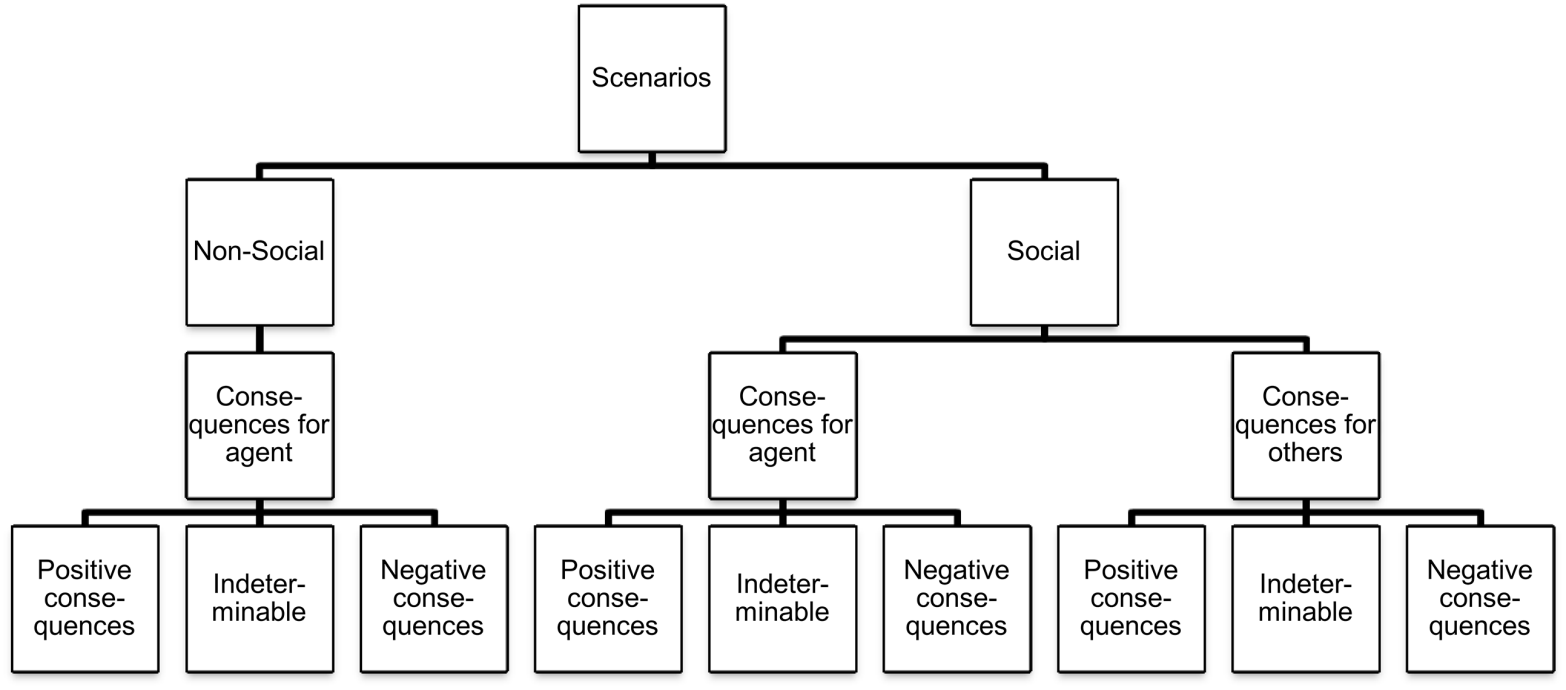
**Coding scheme for scenarios**.

Secondly, the actions described in the scenarios differed according to whether they would likely have good or bad consequences for the agent or for the other people involved. Again, the question arose whether there is a difference between the relative degree of association of each of these two cases with intentional and unintentional actions. This dimension allowed us to test hypothesis 1. In case no consequence was explicitly mentioned in a scenario, coders were instructed to judge what kind of effect the described action usually has, based on their own experience and knowledge. As it is sometimes difficult to determine whether an action has positive or negative consequences, and as a particular consequence might be neutral in valence, we also offered our coders the opportunity to choose the option ‘indeterminable consequences.’ The coders had to follow the decision tree depicted in **Figure 1**. Either they labeled a scenario as ‘non-social’ or ‘social.’ If they labeled it as ‘non-social,’ they only had to determine the goodness of the consequence of the action for the agent. If they instead decided that the scenario belongs to the social category, they had to decide for both the agent and for the other person(s) whether the consequence is good, bad, or indeterminable.

#### Explanation Categories

In order to address hypotheses 2 and 3, we searched through the explanations to find relevant criteria for intentional and unintentional actions. Our analysis methodology was based on the basic principles of Grounded Theory (Glaser and Strauss, 2009). That is, we used qualitative responses as a means to identify concept components that are as close to the actual utterance as possible. Since we did not want to subsume possibly unrelated criteria under a single category based on theoretical assumptions, we first generated a list of key words from the explanations. Key words were merged only when they were evidently synonymous (both semantically and pragmatically). Using dictionaries and logical analyses, synonymous phrases were reduced and global categories were formed. During this procedure, we made sure that semantic content was grouped correctly without losing any significant information. English native speakers were involved in this process. We found criteria of various kinds, including mental states of the agent (e.g., intention, desire), capacities of the agent (e.g., free will, skill), but also features of the action (e.g., norm-violating, being an accident, being a side effect), and the manner in which the action was performed (e.g., actively, automatically, as a routine).

In total, we included 18 category labels for the intentional action explanations: ‘intention,’ ‘decision,’ ‘desire,’ ‘thinking about the action,’ ‘free will,’ ‘control over the action,’ ‘doing something in order to achieve something,’ ‘actively doing something,’ ‘effort,’ ‘knowing how to perform an action,’ ‘knowing about the consequences of one’s action,’ ‘routine,’ ‘awareness,’ ‘normally intentional,’ ‘norm-violating behavior,’ ‘mental capacity,’ ‘skill,’ and the residual category ‘other’.

For the unintentional action explanations, we included 23 criteria, some of which were negations of the criteria for intentional action and some of which were unique to the unintentional action explanations: ‘lack of intention,’ ‘lack of decision,’ ‘lack of desire,’ ‘not thinking about the action,’ ‘lack of control because of external factors,’ ‘lack of control because of internal factors,’ ‘negative effects for the agent,’ ‘not actively doing something,’ ‘effortlessness,’ ‘not knowing how to perform an action,’ ‘not knowing about the consequences of one’s action,’ ‘automatic behavior/ response,’ ‘lack of awareness,’ ‘normally unintentional,’ ‘inattention,’ ‘accident,’ ‘action is based on false information/ assumptions,’ ‘indifference,’ ‘unconsciousness,’ ‘not consistent with the agent’s personality traits,’ ‘feeling regret,’ ‘side effect,’ and the residual category ‘other.’

There is no one-to-one mapping of the intentional and unintentional criteria, i.e., we did not add an opposing version of each item to the other list and vice versa. We refrained from creating such a mapping because this would have increased the number of criteria tremendously, making the coders’ work much more difficult. Also, creating independent lists for intentional and unintentional action criteria allowed us to stay closer to the actual wording used by our participants in these two conditions. Coders were allowed to assign more than one category to each explanation.

#### Coding Procedure

Coding was initially completed by a total of 16 undergraduate Psychology and Cognitive Science students of the University of Osnabrück. Proof of either having studied English for 8 years or being a competent user of English as assessed using language tests is an admission requirement for studying Psychology or Cognitive Science at this university. Besides this formal qualification the coders were interviewed to assess their language competency. They all received course credit for their participation. The material was distributed randomly among them. In an introductory session that lasted for 2 h, they were instructed to strictly follow our coding instructions. They were required to pay close attention to the exact wording of each category description and to select the appropriate categories for the scenarios and explanations. During the following week, they worked on the rest of the data at home, which amounted to an additional workload of about 2–3 h. Consistently, two coders worked on the same material, which enabled us to calculate the inter-observer agreement for each category. The coded data was checked by the authors, to make sure that the coders had followed the instructions accurately. As a result, it became apparent that eight of the 16 coders had not sufficiently followed our directions (more than 10% of the responses were deemed clearly inconsistent with the coding instructions). The same material was then randomly distributed once more among eight new coders.

In general, independent coding of each item, as a basis for later reliability determination, was achieved by the following process: we took the total number of items (scenarios and explanations, respectively) and divided them into subsets, each to be coded by one student. Then we made a second copy of each of these subsets, which was coded by another student. The material of the 786 scenarios was divided into three subsets, each of which was distributed to two coders. As coding of the explanation was viewed to be more time-demanding, the 786 explanations were divided into five subsets, each of which was again coded by two coders. As we came to know during the subsequent analysis that one of these subsets was still faulty (more than 10% of the items were deemed clearly inconsistent with the instructions), we excluded this subset from further analysis. The rest of the coding was deemed to be in accordance with the coding instructions (Supplementary Material). As a result, a total of 644 twice-coded explanations were finally analyzed.

## Results

For each category, we calculated how often it occurred in the total material. We summed the occurrences of the category in the individual subsets that had been given to the coders and, as we had duplicated the material previously, divided the sum by two. Knowing the total frequencies of the categories and the total number of scenarios and explanations, we were able to calculate the ratios presented below.

### Scenarios

In general, our participants were very confident that their scenarios were good examples of intentional or unintentional actions. However, the mean certainty value in the intentional condition (*M* = 2.72, *SD* = 0.51) was significantly higher than in the unintentional condition (*M* = 2.26, *SD* = 1.25), *t*(130) = 4.25, *p* < 0.001, *d* = 0.37.

The ratio of social to non-social context ascription was largely the same for intentional and unintentional action scenarios, χ^2^(1, *N* = 786) = 3.15, *p* = 0.076, *v =* 0.06. For the intentional scenarios, the amount of non-social context categorization was considerably higher (62%) than the amount of social context ascription (38%), χ^2^(1, *N* = 393) = 22.48, *p* < 0.001, *v* = 0.24. The same is true for the unintentional scenarios, where 56% were coded non-social and 44% social, χ^2^(1, *N* = 393) = 5.15, *p* = 0.023, *v* = 0.11. We calculated Cohen’s Kappa (κ) as measure of the inter-observer agreement. A Cohen’s Kappa value of 0 denotes agreement at chance level, while a value of 1 indicates perfect agreement. Hereafter, we will use the common interpretation of Cohen’s Kappa introduced by Landis and Koch (1977) and denote the strength of agreement as slight between 0 and 0.2, as fair between 0.21 and 0.4, as moderate between 0.41 and 0.6, as substantial between 0.61 and 0.8, and as almost perfect between 0.81 and 1. We note that findings that exhibit Kappa values below the middle range of moderate agreement (lower than κ = 0.41–0.6), should be interpreted with caution as they come close to the chance level of the spectrum. Fortunately, the inter-observer agreement along the social/non-social dimension was substantial in the unintentional condition (κ = 0.75) and almost perfect in the intentional condition (κ = 0.84).

**FIGURE 2:**
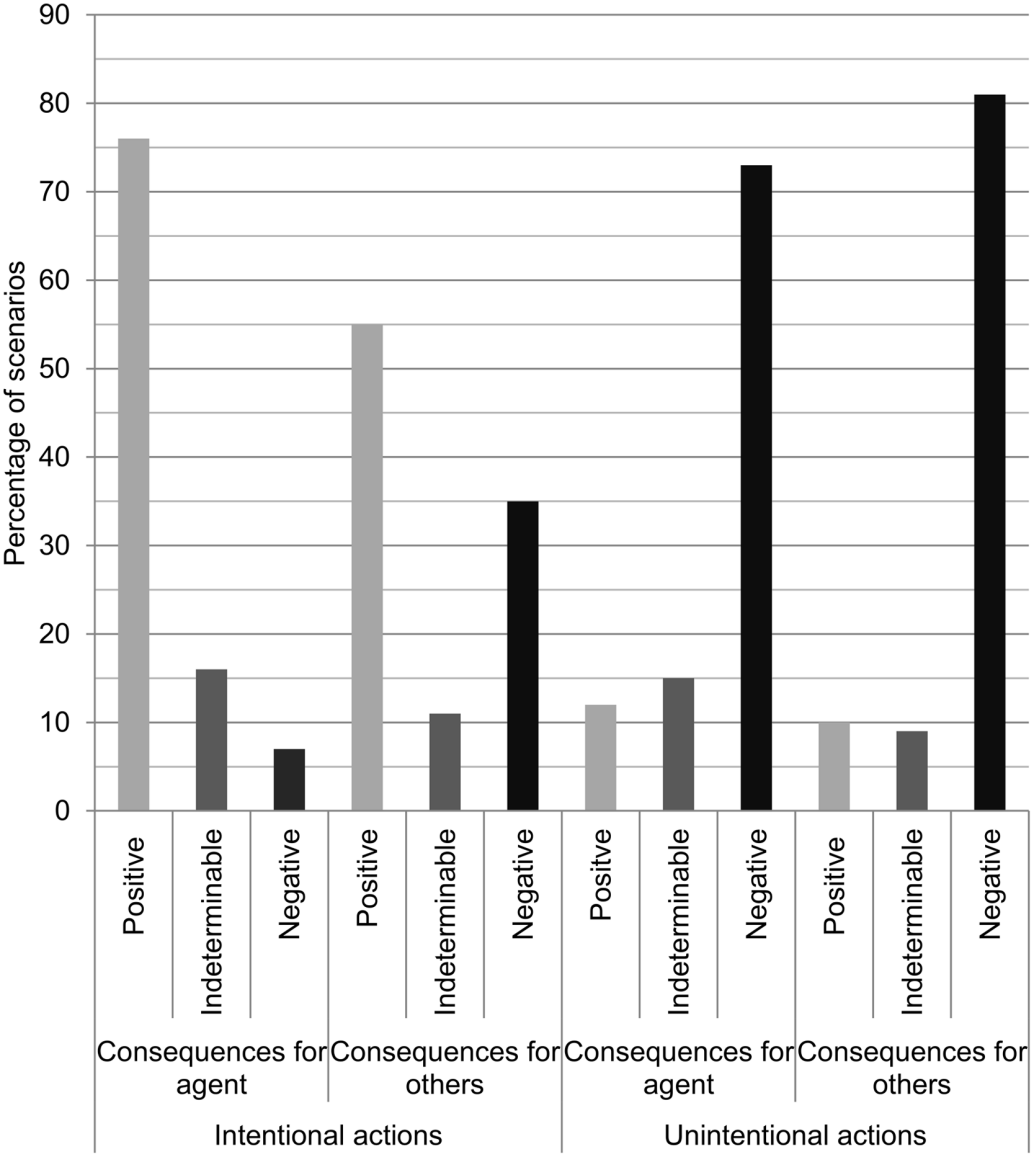
**Valence of the consequences for the agent and for others in scenarios of intentional and unintentional actions**. Bars indicate the percentage of scenarios in which the respective consequence type was present. Note that all scenarios were relevant for coding of the consequences for the agent, but only social scenarios were relevant for the coding of the consequences for others.

The analysis of the consequence dimension confirmed our first hypothesis. Consequences for the agent were evaluated as positive for 76% of the intentional scenarios, while they were evaluated as indeterminable and negative much less frequently (16 and 7%, respectively), χ^2^(2, *N* = 393) = 331.71, *p* < 0.001, *v* = 0.92 (see **Figure 2**). An opposite pattern was observed for the unintentional scenarios. Here, 73% of the scenarios were categorized as having negative consequences for the agent, 15% as having indeterminable consequences, and 12% as having positive consequences, χ^2^(2, *N* = 393) = 282.55, *p* < 0.001, *v* = 0.85. The consequences for agent category in the intentional scenarios revealed only a slight strength of agreement (κ = 0.21) and should thus be interpreted with caution. The consequences for the agent in the unintentional scenarios achieved a moderate strength of agreement (κ = 0.52).

For the social scenarios, the coders chose to characterize 55% of the intentional scenarios as having positive consequences for others, 35% as having negative consequences for others, and 11% as having consequences for others whose valence was indeterminable, χ^2^(2, *N* = 141.5) = 41.51, *p* < 0.001, *v* = 0.54. Because of the comparatively high frequency of negative consequences for others, this distribution is clearly different from the aforementioned distribution of the consequences for the agent in intentional action scenarios, χ^2^(2, *N* = 534.5) = 62.07, *p* < 0.001, *v* = 0.34. Again, a quite opposite effect was demonstrated for the unintentional scenarios. Of these, 81% were coded as having negative consequences for others, while 10% were categorized as having positive consequences, and 9% as having indeterminable consequences, χ^2^(2, *N* = 176) = 181.92, *p* < 0.001, *v* = 1.02. There was a substantial strength of agreement for the consequences for others category in the intentional explanations (κ = 0.71), but only a fair strength of agreement in the unintentional explanations (κ = 0.31).

### Explanations

**FIGURE 3:**
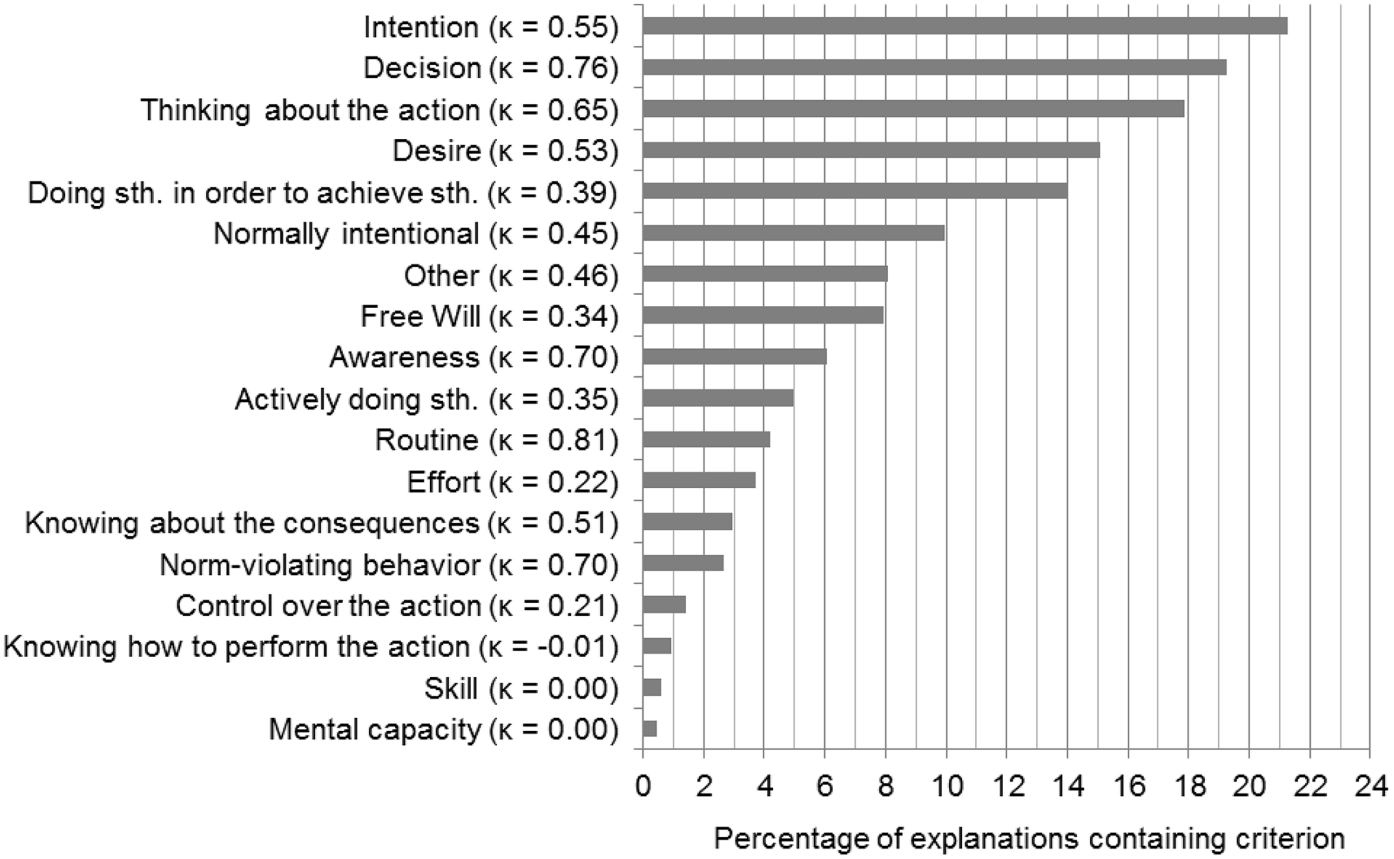
**Categories in descending order of their occurrence in participants’ explanations for why a described action is intentional.** Bars indicate the percentage of explanations in which the respective category was present. Summed percentages exceed 100% as some explanations contained more than one category. Cohen’s Kappa reliability values are noted in brackets behind the category names. Some names are abbreviated versions of the labels used in the coding instructions.

Coders were allowed to choose more than one category per explanation. In regards to intentional action, they most often assigned ‘intention,’ which was present in 21% of the explanations, closely followed by ‘decision’ (19%), ‘thinking about the action’ (18%), ‘desire’ (15%), ‘doing something in order to achieve something’ (14%), and ‘normally intentional’ (10%; see **Figure 3**). This lends initial support to our hypothesis 2. We will further elaborate on this issue in the discussion section.

As these were the most prominent categories, a few more words about how they were defined in the coding instructions are in order. The category ‘intention’ comprised ‘intending something’ and ‘having an intention,’ as well as phrases like ‘doing something on purpose’ and ‘meaning to do something.’ We included references to choice and to options within the category ‘decision.’ ‘Thinking about the action’ was defined as a conscious thought process that precedes the action. Coders were required to choose this category if the explanation referred to relevant planning, deliberation, consideration, premeditation, or preparation in advance of the action. Under the label ‘desire’ phrases like ‘wanting/ wishing to do something,’ ‘feeling like doing something’ or ‘need to do something’ were grouped. As object of desire, both the action itself and the consequences of the action were possible. Examples for phrases belonging to the category ‘doing something in order to achieve something’ included ‘doing something in order to,’ ‘having a reason to do something,’ ‘having an aim/ objective in mind,’ and ‘aiming to achieve a result/ goal.’ If a participant claimed that a particular kind of action is always done intentionally or that the action is impossible to be performed unintentionally the category ‘normally intentional’ was chosen. For example, if a participant explained that tying shoe laces cannot be done unintentionally, this category was applied.

Of these five most mentioned categories, only ‘doing something in order to achieve something’ failed to reach a moderate strength of agreement (κ = 0.39). In general, the strength of agreement observed between coders was as follows: almost perfect (κ = 0.81–0.99) for one category, substantial (κ = 0.61–0.80) for four categories, moderate (κ = 0.41–0.60) for five categories, and fair (κ = 0.21–0.40) for another five categories. Only three categories (‘Knowing how to perform the action,’ ‘Skill’ and ‘Mental capacity’) exhibited agreement at chance level or below (κ <= 0). However, this aspect is only marginally relevant, because these three categories have been applied to very few explanations.

**FIGURE 4:**
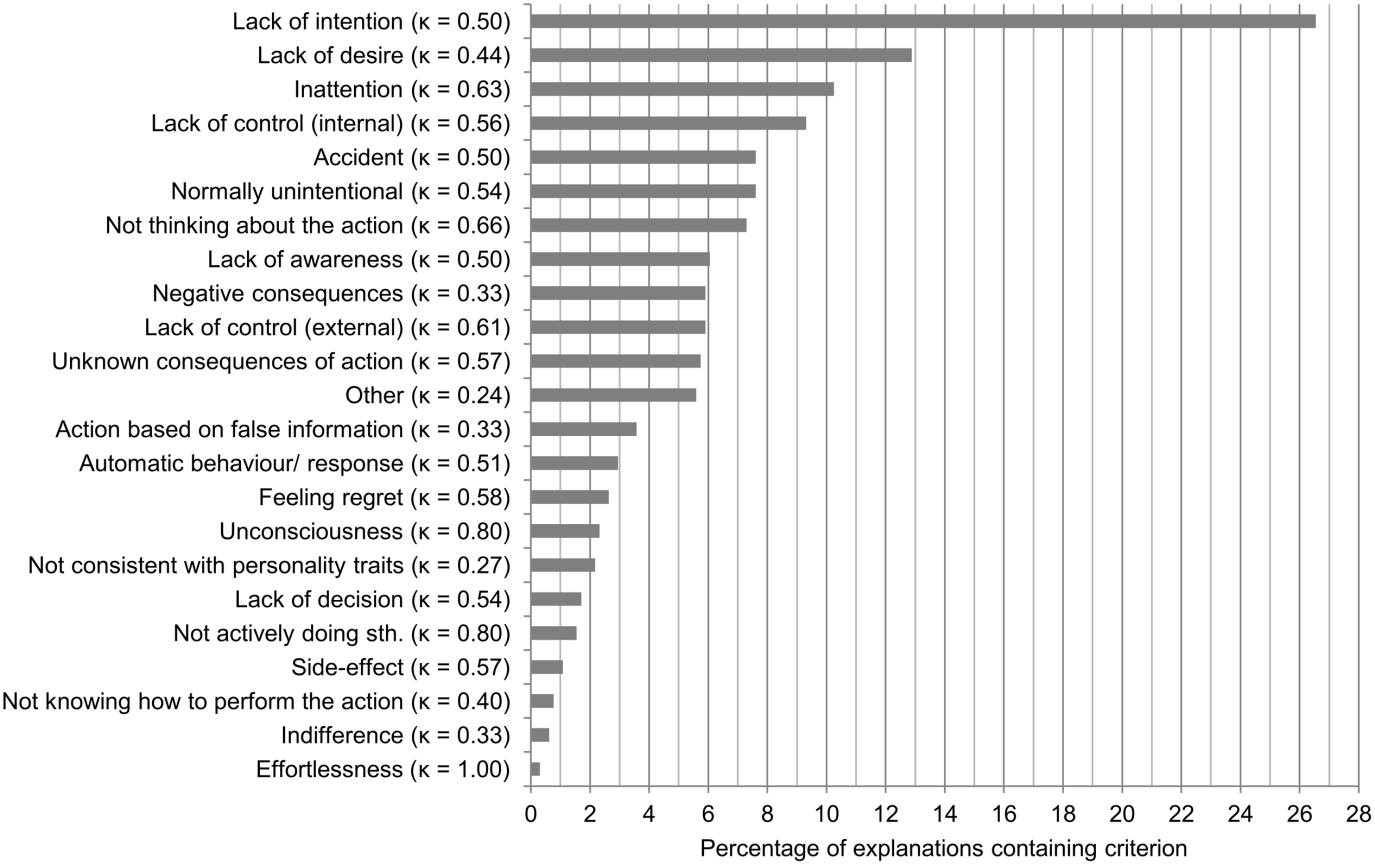
**Categories in descending order of their occurrence in participants’ explanations for why a described behavior is unintentional.** Bars indicate the percentage of explanations in which the respective category was present. Summed percentages exceed 100% as some explanations contained more than one category. Cohen’s Kappa reliability values are noted in brackets behind the category names. Some names are abbreviated versions of the labels used in the coding instructions.

Comparing the criteria mentioned for intentional action with those for unintentional action confirms our third hypothesis, according to which the unintentionality concept differs in its components from the intentionality concept. As already indicated, the category ‘intention’ was found only marginally more often in the explanations for why an action is intentional than the second most frequent category, ‘decision.’ For the explanations of unintentional actions, a quite different picture emerged (see **Figure 4**). Here ‘lack of intention’ (27%) was more than twice as often mentioned as the second most prominent category – in this case ‘lack of desire’ (13%). Following next were ‘inattention’ (10%), ‘lack of control because of internal factors’ (9%), ‘accident’ (8%), and ‘normally unintentional’ (8%). ‘Lack of intention,’ ‘lack of desire,’ and ‘normally unintentional’ were defined as the absence of their above-mentioned counterparts. The label ‘inattention’ was used for behavior that was said to have occurred because the agent acted carelessly or did not attend sufficiently to what she was doing. Following our guideline, so as to stay close to the participants’ responses, we defined two ‘lack of control’ categories. Two quite different sources of lack of control are possible. The source can be external to the agent, e.g., if someone forces her to do something, and the source can be internal, e.g., if the agent has a compulsive disorder or is overwhelmed by her emotions. Apparently, ‘lack of control because of internal factors’ appeared more often in the explanations than external lack of control. Adding up the percentages of the lack of control categories would result in the second most frequent category (15%), which would, however, still be much less frequent than ‘lack of intention’ (27%). Also, simply adding up the percentages of both categories leads to an overestimated result. As coders were allowed to assign several categories to one explanation, they could also assign both kinds of lack of control to one explanation. Thus, by adding up the frequencies presented here, some explanations might be counted twice. Lastly, the definition of ‘accident’ was straightforward. All behavior that was described as happening accidentally or as the result of a mistake was labeled with this category. All these most mentioned categories exhibited at least a moderate strength of agreement between coders (κ >= 0.41). In total, one category showed a perfect agreement between coders (κ = 1), five categories exhibited substantial (κ = 0.61–0.80) agreement, eleven categories moderate (κ = 0.41–0.60) agreement, and six categories fair (κ = 0.21–0.40) agreement between coders.

## Discussion

### Scenarios

The high certainty values confirm that it was easy for our participants to come up with examples of intentional and unintentional actions. Hence, we are reassured that their responses genuinely reflect their understanding of intentionality. However, on average, participants felt more certain about the intentional scenarios than about the unintentional ones. Reviewing the explanations, a possible reason for this becomes apparent. It seems that some participants were aware that seemingly unintentional behavior can still prove to be intentional if certain conditions are met. For instance, one participant came up with a scenario in which the agent is busy at work and so forgets to meet a friend at the cinema. On the face of it, forgetting an appointment is unintentional, but the participant was not so sure, as can be seen by the explanation: ‘He was preoccupied with work and lost track of time. It is a possible (sic) that he didn’t really want to see the movie and therefore allowed himself to lose track of time.’ In this case, the participant’s certainty is lowered, as forgetting the appointment might be intentional under the specific condition that the agent deliberately lost track of time. Another source of confusion might have been that what some participants described was not, in fact, a case of unintentional action, but, rather, of what some philosophers have described as non-intentional action (Chan, 1995). Similarly, some participants might have been unsure about whether their example involves any action at all. Arguably, forgetting an appointment is not an action.

The social/non-social context dimension did not reveal a considerable difference between intentional and unintentional action scenarios. The higher number of non-social contexts than social contexts for both scenario types reflects the fact that situations involving only the agent came more readily into the mind of our participants than situations featuring more than one person. Also, it might indicate that our participants tended to keep their stories simple by focusing on the main protagonist. For both the intentional and the unintentional cases, we found a high inter-coder agreement along this dimension. This shows that the coders had, as expected, no difficulties deciding whether a given scenario involves only the agent or others as well.

The consequence dimension confirmed hypothesis 1, according to which intentional behavior is associated with positive outcomes, whereas unintentional behavior is linked to negative consequences. The consequences for the agent in intentional action scenarios were deemed positive far more often than negative. This finding is not surprising, as it reflects the fact that people do not normally perform an action intentionally that creates disadvantages for themselves. The desire to create positive outcomes for oneself (or to prevent harm from oneself) can arguably be seen as one of the central psychological attitudes of an agent. According to Sripada’s (2010) deep self model, actions that concord with the agent’s core psychological attitudes are likely to be judged intentional. Other accounts of intentionality ascription are less apt to explain the data. For example, the models by Machery (2008) and Uttich and Lombrozo (2010) refer to costs and norm violations, respectively, but the non-social scenarios involving intentional action largely lack these factors. However, one has to bear in mind that the mentioned models were designed in order to account for the asymmetries in side-effect scenarios like the one used by Knobe (2003a) and are thus not meant to provide an account of intentionality ascriptions *per se*. A word of caution is in order, because the coders agreed only slightly on the goodness of consequences for the agent. This is probably due to the difficulty in deciding whether a consequence is indeed good or just neutral. There were many scenarios for which one coder classified the consequences as good for the agent, whereas the second coder classified them as indeterminable. An example of such a scenario is the following one: ‘John was sitting on his couch at home watching TV. He picks up the remote and changes the channel.’ On the basis of the given information, it is indeed hard to say whether it is good for John to change the channel. We instructed the coders to use their ‘experience and knowledge about the world to decide about the goodness of consequences.’ Of course, this leaves considerable room for interpretation. However, although the coders did not always agree on what category to assign, each individual coder classified far more intentional scenarios as having good consequences for the agent than as having bad consequences, indicating that the described effect is robust.

The consequences of intentional actions for others were more often positive than negative as well. Apparently, examples of someone intentionally doing something good for someone else were more easily accessible in the minds of our participants than examples of someone intentionally harming someone else. However, the frequency of negative consequences for others in intentional action scenarios was considerably higher than the frequency of negative consequences for the agent in intentional action scenarios. This is still in line with Sripada’s (2010) concordance principle, because bringing about bad consequences for others may indeed concord with an agent’s central psychological attitudes, if we assume that the agent has a mean character. Take for example this scenario: ‘John intentionally rips out an important part of a book so that his classmate who needs the same material cannot get the information his (sic) needs.’ However, the literature on the Knobe effect provides us with an array of additional, and presumably mutually compatible, explanations for the comparatively higher proportion of scenarios with negative consequences for others than for the agent in the intentional action condition. For example, we can speculate that the common practice of assigning blame by means of ascribing intentionality might have guided participants to come up with scenarios in which the agent harms others (Adams and Steadman, 2004). Moreover, bringing about negative consequences for others usually involves a norm violation (e.g., violation of the norm not to sabotage with others learning materials) and we might assume with Uttich and Lombrozo (2010) that the common link between norm violations and intentionality ascriptions led many participants to come up with cases of norm violations when asked for an intentional action. Finally, one might claim in line with Machery (2008) that actions that incur costs (e.g., negative consequences for other people) in order to reap benefits are commonly associated with intentionality. Although the negative consequences for others were more frequent than the negative consequences for the agent in the intentional action condition, it needs emphasizing that the positive consequences for others outbalanced the negative consequences for others in the intentional action condition. This shows that the link between negative effects and intentionality of an action that has been found in side-effect scenarios such as the ones used by Knobe (2003a) does not generalize to intentionality ascriptions *per se*. In general, people are quite willing to say that an action that did produce a positive outcome for others has been performed intentionally. This confirms our assumption that the results from scenario studies must be interpreted with caution. They can provide insights about which factors can influence the application of a concept, but they certainly do not necessarily inform us about the relative importance of those factors in day-to-day applications of the concept. The coders reached a substantial degree of agreement with respect to the consequences for others in the intentional action scenarios. Apparently, the consequences for others were easier to determine than the consequences for the agent. Presumably, participants made it very plain whether the agent’s action was intended to affect others positively or negatively, but did not state as clearly whether or not the agent benefited from it.

Turning to the consequences of the unintentional actions, we observed a reversed pattern. Here, the negative consequences for both the agent and for others clearly outnumbered their positive counterparts. Most participants came up with stories of accidents and characterized the agent as acting inattentively or as lacking control over the behavior in question. To name just one example: ‘Paul knocks a glass off the table. He’s sitting at the table eating breakfast.’ It is commonsense that people do not normally bring about such effects intentionally. The unintentional action scenarios with effects on others show a similar pattern: ‘Paul was crossing the road and was looking down at the newspaper he was reading instead of where he was going so he bumped into someone by accident and they fell over.’ Again, these findings are in line with Sripada’s (2010) deep self model. One can speculate that accidents are usually at odds with the agent’s core psychological attitude that one should prevent harm to oneself and others, and are thus judged to be unintentional. However, other explanations from the literature on intentional action remain possible. Take for example the pragmatic aspects emphasized by Adams and Steadman (2004). The practice of excusing one’s behavior by saying that it was unintentional might have prompted participants to come up with scenarios featuring bad outcomes. Once again, the observed pattern speaks against the generalizability of the Knobe effect. In Knobe’s (2003a) study, positive consequences (helping the environment) were linked to the judgment that bringing forth these consequences was unintentional. By contrast, we found that our participants link unintentional actions to negative outcomes. We assume that the Knobe effect is specific to side-effect scenarios of a specific kind, but of course few of our day-to-day actions share these features. In any case, the clear prevalence of negative effects in the unintentional scenarios that our participants came up with is striking, considering that there are actually a lot of potentially unintentional every day behaviors without negative effects that people could have mentioned: inattentively listening to music, walking around while being occupied with one’s thoughts, rocking the chair on which one is sitting, etc. The coders’ agreement with respect to the consequences for the for others was comparatively low in the unintentional condition (κ = 0.31). This is once again due to the problem that some coders were more inclined than others to classify the consequences as indeterminable. However, each coder classified the consequences as negative in the vast majority of the cases, which let us assume that this general effect is robust.

### Explanations

#### Components of Intentional Action

Our analysis provides a close-up view of the criteria people associate with the notion of intentional action. Rather than applying a few criteria inferred from theory to the participants’ responses, we followed a bottom-up approach by staying close to their actual statements. An important question that we will address in this section is how our findings relate to the widely held view that intentions, desires, and beliefs are the sole core components of the intentional action concept (e.g., Bratman, 1987; Audi, 1997; Mele, 2009). Also, we will elaborate on other criteria, especially awareness and skill, which have been mentioned in the experimental philosophy literature (Malle and Knobe, 1997).

At first glance, only two of the components included in classic three-way models of intentional action are present in our list of criteria: intention is at the top position in our ranking and desire is at the fourth position. Instead, supporting our hypothesis 2, a range of other criteria are present. We did not even include a ‘belief’ criterion in our coding instructions, as this word and its derivations almost never occurred in our participants’ responses. In fact, it occurred only four times in the 322 intentional action scenarios analyzed, while two of these occurrences were clearly about the participants’ beliefs and not about any belief state of the agent. However, other criteria in our list might reflect what other authors would subsume under the label ‘belief.’ Similarly, there might be criteria in our list that other authors would add to the ‘intention’ and ‘desire’ categories. Hence, it is worthwhile to examine how others define these categories. Here, we will focus on the definitions given by Malle and Knobe (1997, study 2), as our investigation was inspired by their study.

We will begin with intention, as this was the most often mentioned component both in our and in Malle and Knobe’s survey. As already mentioned, in their study, a response had to mention ‘the intention to perform the act, intending, meaning, deciding, choosing, or planning to perform the act’ in order to qualify for the intention category. Interestingly, ‘decision’ was the second most mentioned category in our study. Had we collapsed our ‘decision’ category with the ‘intention’ category, as proposed by Malle and Knobe, this would have led to a huge frequency difference between this merged category and what would then become the second most mentioned category, ‘thinking about the action.’ Note, however, that simply adding up the percentages of the intention and decision categories mentioned in the results would lead to an inaccurate frequency estimate of the merged category, as both categories might have been assigned to single explanations. In addition, references to planning, which we grouped under ‘thinking about the action,’ would probably qualify as ‘intention’ on Malle and Knobe’s account. However, in our view, deciding and planning are conceptually different enough, both from each other and from intending, that it is not warranted to conflate them into one single ‘intention’ category. Although one might argue, in defense of the above account, that planning an action or deciding upon an action usually leads to an implementation intention, we note that this does not imply that the former are equivalent to the latter within peoples’ conception of intentionality. People might, for example, focus solely on the planning or decision aspects of an action while not being aware of the intention aspect of this action when determining whether something was done intentionally. Merging all these aspects into one category renders it impossible to examine whether this is the case. Admittedly, one might also argue that responses that fell under the category ‘doing something in order to achieve something’ are closely linked to intention. This category was chosen if the agent was said to act for a reason or to have a goal in mind and this might indeed imply having an intention. Admittedly, the low coding agreement for the category ‘doing something in order to achieve something’ likely reflects that the coders were confused about whether to assign this category or the category ‘intention.’ As Malle and Knobe kindly provided us with the responses from their study, we are able to report that the word ‘intention’ was mentioned only three times among their subjects’ 159 responses. Nevertheless, their coding resulted in a frequency of 51% for the ‘intention’ component. The low frequency of the actual word ‘intention’ in their data might be due to the participants’ reluctance to give circular definitions. They might have simply considered it obvious and not worth mentioning that an intention is necessary for acting intentionally. Our study design counteracted this confounding factor. As our participants were required to give several explanations, each more elaborate than those elicited by Malle and Knobe, they had enough room to mention a wide range of criteria, including the most obvious. Consequently, we detected ‘intention’ to be the most important component, although we stayed closer to the explicit statements of our participants.

The desire category was assigned by Malle and Knobe if a response mentioned ‘the desire for an outcome or the outcome itself as a goal, purpose or aim.’ This corresponds fairly well with our definition of desire. However, we have to assume that they also assigned the ‘desire’ label to responses that we grouped under ‘doing something to achieve something.’ Even though we acknowledge that someone who does something in order to achieve something is likely to have a desire for what is to be achieved, we think that our principle of staying close to what participants actually mention warrants our distinction. However, we acknowledge that the close connection between ‘doing something to achieve something’ and ‘desire’ might be another factor that contributed to the problematically low coder agreement for the category ‘doing something in order to achieve something.’ Nevertheless, we agree with Malle and Knobe that desire seems to be central to people’s conception of acting intentionally.

As already mentioned, the term ‘belief’ and its synonyms rarely occurred within our participants’ responses. Philosophical models of intentional action frequently emphasize beliefs about how desired outcomes can be brought about, or beliefs about one’s ability to perform a certain action and to produce a certain outcome. This corresponds in our study very closely with the categories ‘knowing about the consequences of one’s action’ and ‘knowing how to perform an action.’ The category ‘knowing about the consequences of one’s action’ has only been mentioned in about 3% of the scenarios and the category ‘knowing how to perform an action’ in about 1% of the scenarios. However, we acknowledge that the conclusion that beliefs play no role in people’s conception of intentional action would be premature. Arguably, although our participants have not explicitly mentioned belief states, this component is likely hidden in the third most often mentioned category, ‘thinking about the action.’ Thinking about an action obviously draws on beliefs, which may include beliefs about the consequences of the action or about one’s ability to perform the action. Accordingly, one can reasonably argue that the belief component is implied in references to this category. Similarly, references to the category ‘doing something in order to achieve something’ might tacitly imply the belief that one can achieve a desired outcome, adding another reason why the coders did not sufficiently often agree on when to assign the ‘doing something in order to achieve something’ category. As a result, we must remain somewhat agnostic about the importance of the belief component in the lay conception of intentional action. The evidence is mixed: People rarely make explicit references to belief states in their explanations for why a given action is intentional, but from a conceptual analytic point of view many of the references they make imply belief states. It might just be that non-philosophers are less used to the talk about beliefs than philosophers and thus rather refer to various kinds of thought processes implying beliefs.

Let us now turn to the question as to how our findings relate to Malle and Knobe’s claim that awareness is a further important component of people’s intentionality concept. Similarly to our characterization of the awareness category, Malle and Knobe assigned this category if a definition mentioned ‘awareness of the act while the person is performing it.’ In contrast to their study, awareness played only a minor role in the responses of our participants. Also, our other categories do not seem to reflect what Malle and Knobe mean by ‘awareness.’ Admittedly, one might argue that thinking about an action usually involves awareness. However, our ‘thinking about the action’ category was clearly defined as encompassing thoughts preceding the action, whereas Malle and Knobe point to awareness of performing the action. Hence, we can conclude that conscious thought before the action but not necessarily awareness during action is part of people’s conception of intentional action.

The agent’s skill in performing an action played almost no role in our participants’ responses. Malle and Knobe speculated that the lack of the skill component in their participants’ definitions of intentional action may be due to the fact that they may have ‘only considered interpersonal behaviors, for which skill can be assumed’ (p. 109). We can rule out this explanation, because in our study participants did consider behaviors that were not interpersonal (what we call non-social scenarios) and still did not mention skill as an important criterion.

In summary, we propose that intention, desire, decisions, and thoughts about actions are indeed central to people’s understanding of intentional action. Belief might well be another central component of the lay conception of intentional action. Although people do rarely refer to belief states when explaining why a given action is an intentional action, belief states are conceptually implied in some very frequently mentioned categories. One might want to object that decisions and thoughts about actions again imply (or are closely linked to) intentions, beliefs, and desires. This may well be true, but we find it important to stress that decisions and thoughts about actions are not reducible to sets of intentions, beliefs, and desires. Thoughts about actions, for example, may not only involve the tokening of beliefs, desires, and intentions, but also reasoning about these states and drawing inferences. Our participants may genuinely think that intentional action requires thought processes, irrespective of the fact that thought processes may draw on beliefs, desires, and intentions of the agent. Further research is needed to investigate this possibility. For example, one could design scenarios that vary the amount of thoughts an agent spends on planning an action, while keeping the desire, beliefs, and intentions of the agent constant, and examine whether peoples’ willingness to say that the agent acts intentionally is affected by this. We acknowledge that the category ‘doing something in order to achieve something’ is problematic as it is closely related to desire, belief, and intention and therefore exhibited only low coder agreement. In fact, ‘doing something in order to achieve something’ may just be another way of saying that something was done intentionally. Although less important than intention, desire, belief, decision, and thoughts about actions, the category ‘normally intentional’ was also quite often mentioned. Admittedly, this category does not inform us about people’s conceptions of intentionality. But it tells us something about a shortcut that people frequently use when making intentionality judgments. If a behavior is immediately perceived as a typical case of intentional action, people do not require any additional evidence, such as information about the agent’s mental states, to make their judgment. For instance, it is commonsense that binding shoe laces is an intentional activity. Interestingly, free will has also been mentioned remarkably often. However, this result must be regarded with caution as the coder agreement for ‘free will’ was comparatively low.

#### Components of Unintentional Action

To our knowledge, with the exception of the current study, nobody has yet examined in detail what aspects people associate with unintentional behavior. The tacit assumption appears to have been that people’s conception of unintentional action is just an inverted version of their conception of intentional action. In line with our third hypothesis, our study indicates that this assumption is not justified. A comparison of our lists of criteria for intentional and unintentional actions (see **Figures 3** and **4**) reveals considerable differences. However, let us first briefly focus on the commonalities.

In each list, the relevant intention category (‘intention’ or ‘lack of intention,’ respectively) occupies the top position. It is also notable that ‘lack of desire’ turned out to be the second most frequently mentioned criterion for unintentional behavior, because its counterpart ‘desire’ is very prominent in the list of categories for intentional action as well. Apparently, desire plays a central role both in peoples’ conception of intentional action and unintentional action. Another category that is present in both lists is ‘normally (un-)intentional.’ Just as in the case of paradigmatically intentional actions, there are behaviors that are so strongly associated with unintentionality that people do not need to search for further criteria in order to make their judgments. Obviously, someone who falls down the stairs does not normally do this intentionally.

Besides these aspects, there are clear asymmetries between both lists. The large frequency difference between the category ‘lack of intention’ and the second most mentioned category within the list of components of unintentional action, ‘lack of desire,’ is remarkable, considering that the category ‘intention’ was not as clearly distinguished from the other categories in the case of intentional action. This may indicate that people assume having an intention to be necessary but not sufficient for acting intentionally. The absence of an intention is seen as a sign of an action’s being unintentional, but the presence of an intention is not enough for an action to qualify as intentional, because additional criteria must be met. As another asymmetry, ‘decision’ was the second most frequently mentioned category for intentional action but ‘lack of decision’ was almost never mentioned in the case of unintentional behavior. Apparently, for our participants a decision foregoing an action is a clear indicator for the intentionality of the action, but a lack of decision does not seem to be central to their conception of unintentional action. Also, the third most frequently mentioned category in the case of intentional action, ‘thinking about the action,’ was much less frequently mentioned in its negated form in the case of unintentional behavior. Instead, criteria like ‘inattention,’ ‘lack of control because of internal factors,’ and ‘accident’ are important when it comes to unintentional action. All these categories indicate that people strongly associate involuntariness with unintentional behavior. By contrast, when asked why an action is intentional, people presumably see no need to mention that the action was no accident, that the agent had control over what he was doing, and that he paid attention to his action.

### Limitations and Objections

A shortcoming of our study is certainly that for some categories the coders did not even achieve moderate agreement (κ < 0.41). With respect to our scenario categories, this was the case for consequences of the agent in intentional action scenarios and the consequences for others in unintentional action scenarios. Consequently, the respective results have to be interpreted with caution. In this study, we instructed the coders to judge what kind of effect the described action usually has based on their own experience and knowledge. This was in line with our general approach of not imposing those preconceptions that we hold as researchers on the coding process. However, future studies could explore how the coding reliability could be increased by giving coders more precise guidance on what is to be coded as a good or bad consequence for an agent and other people affected by the action. With respect to the explanation categories, a higher inter-rater reliability might have been achieved by formulating fewer categories. However, there is a trade-off between the informativeness of the data gained and inter-rater reliability. We decided to conduct an exploratory analysis, which we deem to be high in informative value, because it allows us to compile an exhaustive list of relevant criteria for intentional action that may inform the hypothesis generation for future experimental philosophy research. For example, our finding that decisions and thoughts about actions were frequently mentioned in the intentional action explanations raises the question as to whether intentionality ascriptions are indeed influenced by these factors, independently of their relation to intentions, desires, and beliefs. One may also object that our approach left room for many subjective decisions as to how to formulate the individual categories. However, we think that compiling a list of all keywords mentioned in all responses and merging only those that are logically, semantically, and pragmatically the same has proven a useful method that outperforms approaches presented in previous studies using the free-response paradigm (Malle and Knobe, 1997). Admittedly, researchers using our method will face trade-off decisions between accurately capturing all relevant components and keeping the list of categories short enough to be useful for later interpretation.

A further limitation of our exploratory approach is that it leaves some room for interpretation as to what our participant’s explanations actually reflect. First of all, it is not clear whether the participants deemed those criteria that they mentioned sufficient, necessary, or both necessary and sufficient for acting (un-)intentionally. For example, we may interpret an explanation that contains several components as an exhaustive definition, including several necessary and jointly sufficient conditions, or as a loose set of several conditions that may be taken to be individually sufficient but not necessary for acting intentionally. We cannot resolve this interpretation problem at this point. All we can say is that when we aggregate the participants’ responses some criteria turn out to be more important than others. We leave it to future experimental philosophy research to decide whether people think of these individual criteria as necessary, sufficient or both necessary and sufficient conditions for acting intentionally. Secondly, it must be noted that our participants’ responses are fallible guides to what factors actually influence intentionality ascriptions. Their responses reflect what they think are reasons for saying that a given action is intentional, but of course the actual psychological processes that guide their intentionality ascriptions might be blind to (some of) these reasons or sensitive to other factors. For example, it is possible that people are not aware of all those factors that drive their intentionality ascriptions, or that they name for pragmatic reasons only some of these factors.

Relatedly, one may object that participants might simply have considered some explanations for why an action is intentional or unintentional overly obvious and thus not worth mentioning, as suggested by Gricean maxims of conversation (Grice, 1975). Consequently, our results would not reflect the most important components of people’s conceptions of intentional and unintentional action, but rather those components that our participants consider most informative. This possible shortcoming of studies relying on free-response explanations has been discussed by Böhm and Pfister (2015) based on a variety of similar studies (Hilton, 1990, 2007; Slugoski et al., 1993; Hilton and Slugoski, 2001). However, if it were the case that our participants did not mention important criteria due to their low informative value, they should have also been reluctant to name intention as a criterion for intentional action. After all, it is most obvious that intention is linked to intentional action. As we have seen, ‘intention’ actually turned out to be the most often mentioned category, speaking against this view.

Lastly, one might object that our findings have no implications for philosophical analyses of intentional action. After all, philosophers have, in contrast to our participants, considerable expertise in analyzing concepts. Our participants might simply be mistaken about what conditions must be met for acting intentionally, while philosophers might be better trained for this. Like the expert in physics, who should not consult folk physics in order to learn something about magnetism, one might argue that the philosopher should not rely on lay persons’ responses in order to learn something about intentional action. However, on a closer look, there is an important difference between these cases. Whereas ‘magnetism’ is a theoretical term rarely used in ordinary conversation, ‘intentionality’ is a term that is common to peoples’ linguistic practices. Hence, it would be odd if philosophers called something ‘intentional action’ that is largely unrelated to people’s use of the term (Knobe and Nichols, 2008). At the very least, philosophers who put forward a model of intentional action that is not in accordance with laypersons’ conceptions should be able to explain why their model differs from the general use of the term.

## Conclusion

In light of our results, we are convinced that our exploratory method of probing peoples’ conception of intentionality is a valid and important addition to the ordinary vignette approach used in experimental philosophy and social cognition research. Rather than asking our participants for their conceptual intuitions regarding artificial scenarios, our participants could construe their own scenarios and give their own explanations for why they think that a given action is intentional or unintentional. Consequently, our investigation was largely driven by the actual responses of participants, rather than by previous theoretical assumptions. The analysis of the given scenarios allowed us to detect a clear pattern in the valence of the consequences linked to intentional and unintentional actions. People associate unintentional actions predominantly with bad outcomes for all persons involved and link intentional actions more strongly to positive outcomes, especially concerning the agent. Moreover, we were able to extract from our participants’ explanations a range of components that they link to intentional action. These are most notably intentions, desires, decisions, and thoughts about actions. We can also speculate that beliefs play an important role in peoples’ understanding of intentional action. Our participants did not explicitly refer to belief states in their explanations, but some of the categories that they referred to conceptually imply belief states. Although empirical evidence of how non-philosophers apply the concept of intentional action cannot falsify deviating philosophical analyses of that concept, we think that a convincing conceptual analysis should take into consideration people’s actual usage of the concept in question. Lastly, we showed that people’s conception of unintentional action is not just an inversion of their conception of intentional action. In addition to lack of intention and lack of desire, unintentional behavior is strongly linked to inattention, lack of control, and accidents.

## Conflict of Interest Statement

The authors declare that the research was conducted in the absence of any commercial or financial relationships that could be construed as a potential conflict of interest.
