# Supplementary material for: Investigating conceptions of intentional action by analyzing participant generated scenarios
Source: Front Psychol. 2015 Nov 5;6:1630. doi: 10.3389/fpsyg.2015.01630 (PMC4633502; doi:10.3389/fpsyg.2015.01630)
Supplement: Supplementary file 1 [file Data_Sheet_1.DOCX]

***Supplementary Material***

**Investigating conceptions of intentional action by analyzing participant generated scenarios**

**Alexander Skulmowski^1^*, Andreas Bunge^2^, Bret R. Cohen^3^, Barbara A. K. Kreilkamp^4^, Nicole Troxler^5^**

^1^E-Learning and New Media, Institute for Media Research, TU Chemnitz, Chemnitz, Germany

^2^Department of Philosophy, University of Nottingham, Nottingham, United Kingdom

^3^Institute of Cognitive Science, University of Osnabrück, Osnabrück, Germany

^4^Department of Molecular and Clinical Pharmacology, Institute of Translational Medicine, University of Liverpool, Liverpool, United Kingdom

^5^Institute of Psychology, University of Osnabrück, Osnabrück, Germany

*** Correspondence:** Alexander Skulmowski, E-Learning and New Media, Institute for Media Research, TU Chemnitz, Straße der Nationen 12, Chemnitz, 09111, Germany.

alexander.skulmowski@phil.tu-chemnitz.de

**Supplementary Data**

**A. Coding Instructions**

This appendix displays the complete coding instructions used in our study.

**A.1 Introduction**

Our study group examines the folk notion of intentional action and unintentional behavior. To be more precise, we are interested in the reasons / criteria that people mention or have in mind when they conclude that a certain action is intentional or not. For that reason we asked our participants to first come up with scenarios including intentional actions and secondly to write down scenarios that include unintentional behavior. We encouraged them to give reasons / explanations for why they think the behavior just described is or is not intentional.
Your work material consists of some scenarios and explanations for intentional actions and unintentional behavior that our test subjects gave. The scenarios and explanations for intentional actions are clearly separated from the scenarios and explanations of unintentional behavior.
Every explanation refers to exactly one scenario. We formed two different groups of “coders” (Group A and Group B). Depending on the group you have been assigned to, you will get a different task. The fact that the word “Group” is used here does not imply that you should work together with the others in your group. On the contrary, you should work alone.

**A.1.1 Task of Group A: Classifying Scenarios**

Our participants were asked to come up with scenarios in which an agent acts intentionally or unintentionally. Your task is to determine the context of those scenarios (e.g. specifying how many persons are involved in the mentioned scenario). In order to do that, we provided you with a set of categories which are specified in detail in section 2. Thus, for each scenario, you just have to assign some of the provided categories. How you apply the provided categories correctly, is also explained in section 2. Section 3 is not relevant for you!

**A.1.2 Task of Group B: Classifying Criteria**

Our participants were asked to come up with scenarios in which an agent acts intentionally or unintentionally. Your task is to determine the criteria people mention in the given explanations for why they think that the action described in the scenario is intentional or unintentional. In order to do that, we provided you with two distinct sets of categories: The first set relates to the mentioned criteria for intentional actions, whereas the second set relates to the mentioned criteria for unintentional behavior. Your task is to choose those categories from the given pool which describe the mentioned criteria for intentional or unintentional behavior best. More specific instructions for coding the explanations, including detailed specifications of the categories, are given in section 3. Section 2 is not relevant for you!

**A.2 Instructions for Coding the Context of the Scenarios (Group A)**

As already mentioned, participants were asked to come up with scenarios in which an agent acts intentionally or unintentionally. Please code the given scenarios according to the following instructions. Please note that categories are always depicted and explained in the following format:

**Name of the category**

*Abstract explanation of the category or specification of how the category should be used*

- A typical aspect which falls under this category.
- Another typical aspect which falls under this category.

**A.2.1 Distinguishing Between “Social” or “Non-social”**

Please decide first, whether only the agent (John or Paul) or also other persons (including animals) are mentioned in the scenario. The two categories are as follows:

**Non-social**

*No other person than the agent is mentioned in the scenario.*

- Paul drove to the post office to ship the package.
- Paul is walking along the sidewalk.

**Social**

*Besides the agent there is at least one other person (or animal) explicitly mentioned in the scenario.*

- Paul is bumping into an old lady on the train.
- Paul is sending a parcel to his girlfriend.

**A.2.2 Consequences of the Action**

If you code a scenario as non-social, please also indicate whether the described action or behavior has a positive or negative impact on the agent’s well-being. Do the same for the scenarios that you coded as being social, but in this case we would like you to decide additionally whether the action has a positive or negative impact on the well-being of the other person(s) mentioned.

In most scenarios it will not be stated very explicitly whether the consequences are good or not. Please use your experience and knowledge about the world to decide about the goodness of consequences. For example, if the scenario is “John makes a sandwich”, you should choose the category “good consequences”, since normally people make sandwiches because they are hungry.

If you really cannot tell whether the consequences are positive or negative or if you think that the consequences are neutral, you can choose the option “indeterminable”. However, avoid excessive use of this option.

Please note that we are interested in the immediate consequences of an action, not the long-term effects of it. A little example from our pool of scenarios might help to illustrate this point:

*“John is a little boy who doesn't like to share his toys. One day his friend came over and played with one of his toys. John intentionally hit him and took the toy back.”*

Here, the immediate consequence of John’s action is good for him: He gets his toy back. Of course one might wonder whether such a behavior will have bad consequences for John in the long run, but this is not what we are interested in. In the following paragraph you will find explanations and examples for each of the consequence categories.

**Good Consequences for the Agent**

*The agent’s action has a considerable positive impact on the agent’s well-being.*

- John is watching TV at home and feels hungry, he goes to the shop and buys bread and cheese, comes home and makes a cheese sandwich to eat.
- John went to the bathroom because his bladder was about to burst and he had to use the toilet.

**Consequences for the agent are indeterminable**

*The agent’s action has neither a good or negative impact on the agent’s well-being or it is not decidable whether the consequences are good or bad.*

- John goes to the parent teacher meeting.
- Everyday actions: putting on shoes, answering the phone, walking to the train station, watering plants and taking a shower.

**Negative consequences for the agent**

*The agent’s action has a negative impact on the agent’s well-being.*

- Paul fell asleep before setting his alarm and therefore overslept the following day which made him very late for his meeting.
- John wants to remove a hornets’ nest from the tree outside his front door. He pours lighter fluid on the nest and intentionally sets it on fire. As a result, his home also catches fire and burns to the ground.

**Good consequences for others**

*The agent’s action has a positive impact on others’ well-being.*

- John regularly goes to church. One Sunday his church collects a special offering for a family whose house has recently burned down. He gives some thought, and decides he can spare $20 to give to the family, so he donates that much. $20 is not an insignificant amount of money and it is a meaningful sacrifice*.*
- John is sitting in the office and a colleague walks in. John is asked to help prepare a presentation for an important client. John knows the client well and decides to help his colleague.

**Consequences for others are indeterminable**

*The action has neither a good or negative impact on other’s well-being or it is not decidable whether the consequences are good or bad for others.*

- John is eating ice cream. He is at home, watching a movie with his wife and enjoying a delicious bowl of “Ben and Jerry's chocolate chip cookie dough ice-cream”.
- Paul arrives in Germany, his whole life having previously lived in New Zealand. He goes to the supermarket, and, without thinking, asks the checkout girl, "How are you?"

**Negative consequences for others**

*The action has a negative impact on other’s well-being.*

- Paul has stopped to think about what else to buy in the supermarket. He is blocking the alley, holding up other shoppers.
- Paul is unintentionally annoying Shelly by sending documents to the printer while she is on the phone at her desk next to the printer.
- Paul is serving wine to his dinner guests. While leaning across the table to pour the final glass he accidentally knocks his own glass over, spilling the red liquid all over a female guest's white dress! Paul is devastated...not to mention what the female guest is feeling!

**A.3 Instructions for Coding the Criteria for the Explanations (Group B)**

For each of the two types of behavior (i.e. intentional and unintentional) you find a separate set of categories which may describe some of the reasons / criteria which are mentioned in the explanations. The set of categories for the intentional actions is described in detail in section 3.1 whereas the set of categories for unintentional behavior can be found in section 3.2.

For every individual explanation your task is to choose those categories from the given pool which describe the mentioned criteria for intentional or unintentional behavior best. Please read the following specifications, explanations and examples carefully:

1. With the term “criteria” we mean aspects which are explicitly mentioned in the given explanation. So if, for example, the explanation is *“The action X was intentional because John decided to do X”*, then you choose the category “Decision” from the set of categories. But if the explanation would be “*The action was intentional because John consciously decided to do X”,* you choose the category “Decision” as well as “Awareness”, since in this case two aspects are mentioned explicitly in the explanation. From both examples it may be possible to conclude or speculate that John may also have the desire to do X, since he decided to do X. This may tempt you to choose the category “Desire” as well. But as mentioned above, we are only interested in those aspects which are explicitly mentioned and “Desire” is an aspect which is not explicitly mentioned – even if it may be implied or can be concluded. Therefore “Desire” should not be chosen in both examples.
2. For each criterion or aspect which is mentioned in an explanation you are allowed to assign exactly one category; so you are allowed to assign as many categories as criteria / aspects are mentioned in the explanation. For example, if the explanation is “*The action X was intentional because John knows how to perform X”*, you may be tempted to choose the category “knowledge about action” as well as “skill (to perform the action)”. But since there is only one aspect mentioned in the explanation above (i.e. “knowing how to perform X”), you are only allowed to chose one category – and in this case the category “knowledge about action” is the better choice. But if the explanation would be “*The action X was intentional because John knows how to perform X and has decided to do X”*, you would be allowed to choose exactly two categories, since two aspects were mentioned in the explanation (i.e. “knowing how to perform X” and “having decided to do X”). So in this case, the categories “Knowledge about action” and “Decision” may be chosen.
3. If you encounter an aspect which you think cannot be classified by using one of the provided categories you should choose the category “other”. We made a lot of effort to provide categories which hopefully cover all aspects mentioned in the explanations. So please choose the category “other” only if you are pretty convinced that the aspect in question cannot by subsumed by any of the provided categories.

**A.3.1 Categories for Criteria of Intentional Action**

Below you find the categories which you should please use for categorizing those aspects of the given explanations which are mentioned as a criterion criteria / reason for an action to be called “intentional”. The categories are depicted and explained in the following format:

**Name of the category [Number of category]**

*Abstract explanation of the category or specification of how the category should be used*

- A typical aspect which falls under this category.
- Another typical aspect which falls under this category.

**Intention [1]**

*The agent is said to have an intention to perform the action in question. Synonymous phrases are "doing sth. on purpose" and "meaning to do sth."*

- Intending (to do) sth.
- Having an intention / intent
- Doing sth. on purpose
- Meaning to do sth.

**Decision [2]**

*The agent decides to perform an action. The decision may involve thinking about alternative actions/outcomes. The decision aspect is emphasized. Look out for key words such as decision, choice and option.*

- Decision to do X
- Choose / decide to do sth.
- Action being the result of a decision

**Desire [3]**

*a) The agent desires to perform an action. Please note that in some cases the agent might feel only a weak desire to perform the action in question, but in other cases a certain need / urge / inner force might underlie the performance of an action. Both possibilities should be considered here.*

- Wanting to do sth.
- Wishing to do sth.
- Feel like doing sth.
- Need to do sth.

*b) The agent desires the consequences of the action in question.*

- Desiring sth.
- Wanting the consequence of an action

**Thinking about the action [4]**

*The action is the result of a conscious thought process that precedes the action, e.g. planning to do something. The action is the result of perception (recognition of a situation) and cognition (thinking about what needs to be done).*

- Action was planned before
- Action typically involves planning
- Thinking before acting
- Consideration
- Deliberation
- Preparation
- Premeditation

**Free will [5]**

*The agent's action is stated to be a result of his free will / autonomy.*

- Acting of one's own free will
- Being able to act differently
- Action not being mandatory
- No element of external force or pressure
- Having the ability to refrain from an action

**Control over the action [6]**

*The aspect of the agent's control over an action is emphasized to mark the action as intentional.*

- Being in control

**Doing something in order to achieve something [7]**

*The action is performed in order to cause a certain result. Since the agent has a reason in mind to perform the action in question, it is assumed that he does what he does intentionally.*

- Doing sth. in order to
- Having a reason to do sth.
- Having an aim / objective in mind
- Aiming to achieve a result / goal

**Actively doing something [8]**

*The agent is said to be doing something actively. Look out for the keywords "active“ and "actively“.*

- Doing sth. actively
- Actively trying to do sth.
- Being active

**Effort [9]**

*Since the action in question requires some kind of effort from the agent, it is assumed that he would not perform this task unintentionally. Instead the agent's willingness to perform the action even though it requires effort is taken as a sign of his determination.*

- Action being time-consuming
- Action being tedious
- Action being a complex task
- Action requiring effort (physical, intellectual, financial, etc.)
- Action involving facing difficulties (in order to reach the goal)

**Knowing how to perform an action [10]**

*The agent knows how to perform the action or appropriate contexts / situations for performing it.*

- Knowing how to do sth.

**Knowing about the consequences of one's action [11]**

*The agent has foreknowledge about possible consequences of his action.*

- Knowing the consequences of one's action
- Knowing what would happen
- Results of an action are in line with the agent's expectations

**Routine [12]**

*The action in question is performed as (a part of) a routine. The action can also be described as a habit or an action which is typical for the agent.*

- Action being part of the agent's daily routine
- Action being an example of typical behavior
- Action being a habit

**Awareness [13]**

*It is stressed that the agent is aware of what he is doing. He consciously performs the action in question. Look out for keywords such as "conscious" and "aware".*

- Being aware of what one is doing
- Consciously doing sth.
- Action being a conscious action

**Normally intentional [14]**

*It is either said that this kind of action is normally done intentionally or that the action in question cannot be done unintentionally.*

- Action is impossible to be performed unintentionally or accidentally
- Action is always performed intentionally
- Action is very likely to be performed intentionally

**Norm-violating behavior [15]**

*Since the agent violates a norm by performing the action, it is assumed that he is acting intentionally.*

- Carrying out an action while knowing that it is unjust or morally bad
- Action involves knowingly breaking the law or rules

**Mental capacity [16]**

*The agent is clearly competent and not affected by a psychological state that prevents him from making responsible decisions.*

- Being in sound mind
- Being capable of making decisions

**Skill [17]**

*The agent has the necessary skills and abilities to perform the action in question.*

- Being skilled to perform a particular action
- Being able to do sth.

**Other [18]**

*If there is an aspect that cannot be subsumed under these categories, please state it here.*

- 1. **Categories for Criteria of Unintentional Behavior**

Below you find the categories which you should use for categorizing those aspects of the given explanations which are mentioned a criterion / reason for a behavior to be called “unintentional”. The categories are depicted and explained in the following format:

**Name of the category [Number of category]**

*Abstract explanation of the category or specification of how the category should be used*

- A typical aspect which falls under this category.
- Another typical aspect which falls under this category.

**Lack of intention [1]**

*The agent is said to have no intention to perform the action in question.*

- Not intending / not having an intention to do sth.
- Not doing sth. on purpose
- Not meaning to do sth.
- Doing sth. different than intended

**Lack of decision [2]**

*The agent is not making a decision to perform the action in question.*

- Not choosing to do sth.
- Not deciding to do sth.

**Lack of desire [3]**

*a) The agent does not desire to perform the action in question.*

- Not wanting to do sth.
- Not wishing to do sth.
- Not desiring to do sth.
- Wanting to do sth. different
- No interest in perfoming an action

*b) The agent does not desire the outcome of the action in question.*

- Not wanting sth.
- Not wishing sth. to happen
- Not achieving what one intended to achieve
- No interest in achieving a consequence
- Wanting sth. different to happen

**Not thinking about the action [4]**

*The action is not the result of a conscious thought process that precedes the action. The agent did not plan to perform the action in question.*

- Not planning to do sth.
- Not thinking about sth.
- Doing sth. without thinking
- Action being unplanned

**Lack of control because of external factors [5]**

*Due to external factors the agent has insufficient control over the situation.*

- Being forced to do sth.
- Being unable to do sth.
- Having no alternative options on which to act
- Circumstances (e.g being in a hurry) prevent that the action could be performed properly

**Lack of control because of internal factors [6]**

*The agent is not restricted by external forces but still not able to sufficiently control his own behavior.*

- Lack of control over one's body
- Temporary loss of control, e.g. due to alcohol consumption
- Being overwhelmed by one's emotions
- Reacting impulsively

**Negative effects for the agent [7]**

*Since the consequences of some behavior are negative for the agent, it is assumed that the agent would not be motivated to perform the action in question intentionally.*

- Action being unpleasant
- Outcome of an action is valued negatively by the agent
- The agent does not enjoy to do sth.

**Not actively doing sth. [8]**

*The agent is said to not be doing something actively. Look out for the keywords “active” and “actively”.*

- Not doing sth. actively
- Not actively trying to do sth.
- Being passive

**Effortlessness [9]**

*Since the behavior in question does not require any kind of effort from the agent, it is assumed that he could perform this action unintentionally.*

- Action not requiring any effort
- Action being easy to perform
- Achieving a result very easily

**Not knowing how to perform an action [10]**

*The agent does not know how to perform the action in question.*

- Not knowing how to do sth.

**Not knowing about the consequences of one's action [11]**

*The agent does not know about or is not aware of possible consequences of his action.*

- Not knowing that sth. would happen
- Expecting sth. different would happen
- Being surprised by what happened
- Being unable to anticipate what would happen

**Automatic behavior/response [12]**

*The described action is an automatic behavior of the agent and thus assumed to be performed involuntarily.*

- Action being a habit
- Action being a reflex
- Action being an automatic bodily response

**Lack of awareness [13]**

*The agent is said to be unaware of what he is doing.*

- Not realizing what one is doing
- Unconsciously doing sth.
- Being subconsciously biased

**Normally unintentional [14]**

*It is either said that this kind of behavior is normally unintentional or that the action in question cannot be done intentionally.*

- Behavior is normally unintentional
- Behavior is very likely to be unintentional
- Sth. is impossible to be done intentionally

**Inattention [15]**

*The behavior was said to have occurred because the agent acted carelessly and/or did not attend sufficiently to what he was doing.*

- Not paying attention to what one was doing
- Acting carelessly
- Being distracted
- Not taking care of one’s actions

**Accident [16]**

*Some action happens accidentally.*

- Doing sth. by mistake
- Sth. happens by chance

**Action is based on false information / assumptions [17]**

*The agent is not aware of the fact that he is missing some important information and/or that he is making some wrong assumptions.*

- Being unaware of a key detail of the situation
- Missing some important information
- Making a wrong assumption about some circumstance

**Indifference [18]**

*The agent does not care what to do because different options have equal value for him.*

- Being indifferent about an action
- Not caring what to do

**Unconsciousness [19]**

*The agent performs the action in question while being unconscious.*

- Being unconscious
- Being asleep

**Not consistent with the agent's personality traits [20]**

*Since the action in question is incongruent with the character or values of the agent, it is assumed that he would not be willing to perform this action intentionally.*

- Not being the type of person doing X

**Feeling regret [21]**

*It is assumed that the agent's behavior was unintentional because he feels regret.*

- Regretting one's actions
- Apologizing for one's actions
- Feeling embarrassed because of one's own behavior
- Having a bad conscience because of one's actions

**Side-effect [22]**

*The described outcome is not itself the goal the agent wanted to achieve. Instead, it is either merely a secondary consequence of the agent's action or an intermediate step in an action sequence.*

- Doing sth. solely in order the achieve sth. else
- Doing sth. is an intermediate step and not the agent’s primary focus (e.g. opening a door in order to enter a room)

**Other [23]**

*If there is an aspect that cannot be subsumed under these categories, please state it here.*
